# Combined endoscopic-percutaneous approach for magnetic compression anastomosis in post-transplant anastomotic biliary strictures

**DOI:** 10.1007/s00330-025-11634-w

**Published:** 2025-04-27

**Authors:** Emre Ünal, Ümran Esen, Aycan Uysal, Türkmen Turan Çiftçi, Devrim Akinci, Erkan Parlak

**Affiliations:** 1 Department of Radiology, Faculty of Medicine, Hacettepe University, Ankara, 06100 Turkey; 2 Department of Gastroenterology, Faculty of Medicine, Hacettepe University, Ankara, 06100 Turkey

**Keywords:** Liver transplantation, Anastomosis, Bile duct obstruction, Magnetics, Stents

## Abstract

**Objectives:**

To investigate long-term outcomes of combined endoscopic-percutaneous methods for endoscopically unmanageable anastomotic biliary strictures in living donor liver recipients.

**Materials and methods:**

This retrospective single-center study included 144 patients referred for biliary stricture between November 2017 and May 2023. Eighty-eight patients (leak = 8, non-anastomotic stricture = 3, treatment refused = 12, successful ERCP = 65) were excluded. Patients initially underwent percutaneous biliary drainage. Patients for whom percutaneous intervention was successful in traversing stricture were followed up with fully-covered self-expandable metallic stents and/or plastic catheter stents. However, in case of failure, magnetic compression anastomosis (MCA) was performed.

**Results:**

A total of 56 patients (mean age, 59 years ±11; 35 men) comprised the study group. Percutaneous intervention was successful in traversing the stricture in 26/56 patients. Among the remaining 30 patients, 26 were eligible for MCA, which was performed successfully in 24 patients (92%). The mean duration from magnet placement to internalization was 7.71 ± 2.77 days (95% CI: 6.54–8.88). Altogether, in 47 patients (24 of whom underwent MCA), percutaneous drains were removed following biliary stenting. The mean follow-up was 1082.5 ± 668.2 days (95% CI: 907.49–1257.51). In 19 patients (40%), recurrent stricture was evident at ERCP during a median follow-up of 90 (IQR: 60–210) days following stent removal. The recurrent stricture rate following MCA (*n* = 6/24) was significantly lower compared to patients in whom MCA was not performed (*n* = 13/23; *p* = 0.026). Overall, stent type had no significant effect on patency (*p* = 0.189).

**Conclusion:**

Percutaneous biliary procedures are essential for endoscopically unmanageable post-transplant anastomotic biliary strictures. MCA seems to provide higher patency rates even in patients with total biliary occlusion.

**Key Points:**

***Question*** *What steps can be taken when endoscopy fails in the treatment of post-transplant anastomotic biliary strictures?*

***Findings*** *Percutaneous biliary access and magnetic compression anastomosis can be applied to increase graft survival in the setting of endoscopically unmanageable post-transplant biliary strictures.*

***Clinical relevance*** *Impassable biliary obstructions are unfortunate complications and not uncommon in liver transplant recipients. Magnetic compression anastomosis is an alternative minimally invasive method of treatment for complete biliary occlusions.*

**Graphical Abstract:**

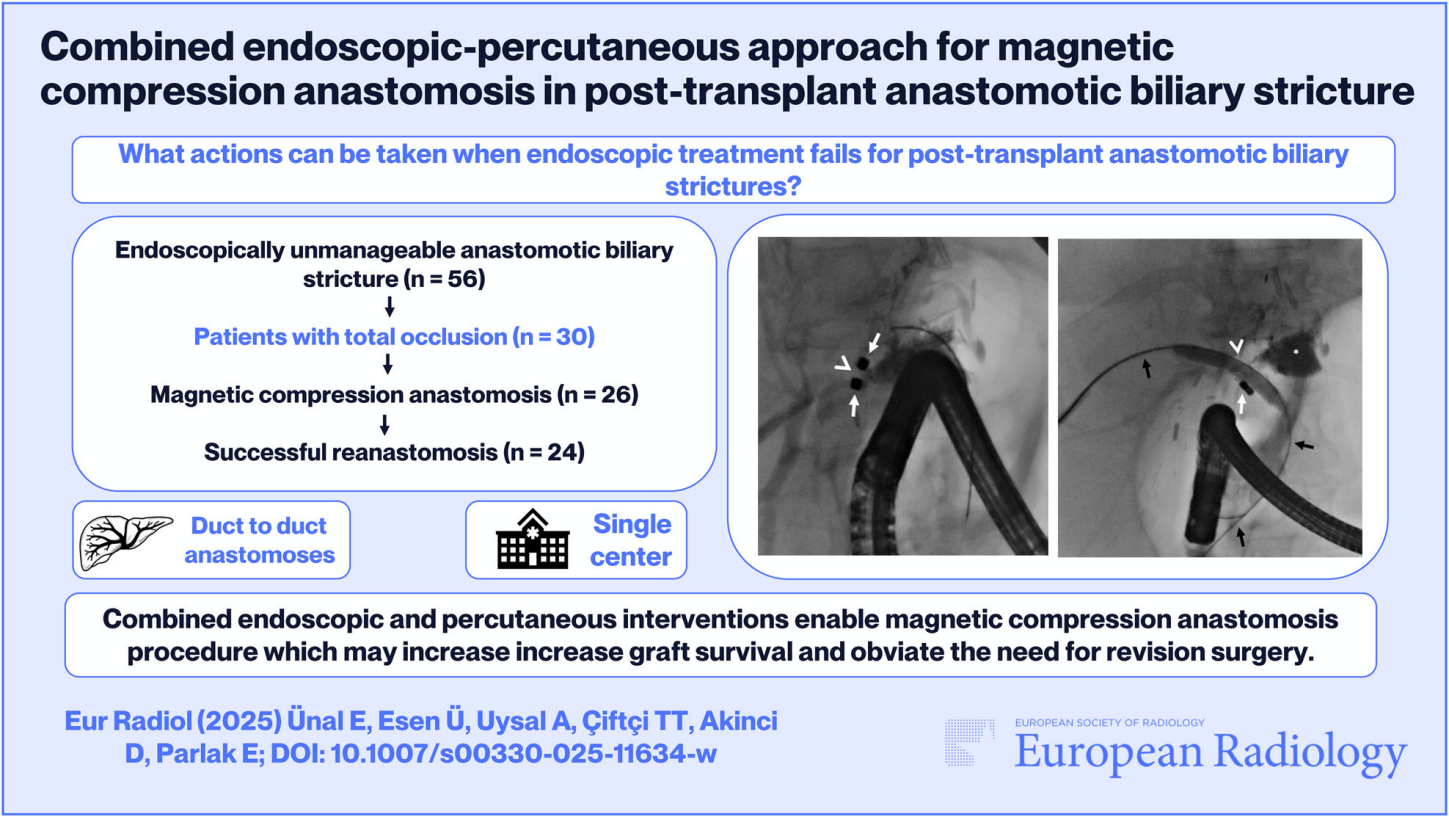

## Introduction

Liver transplantation is a life-saving procedure for patients with end-stage liver disease, acute liver failure, and primary liver cancer [1, 2]. Biliary complications account for the majority of morbidity and mortality following liver transplant. Living donor liver transplantation (LDLT) recipients are more prone to biliary complications compared to deceased donor liver transplantation, due to surgical challenges encountered in LDLT [2]. In addition, treatment of post-LDLT biliary complications can be more challenging, considering multiple anastomoses and the small size of biliary ducts [1]. Early diagnosis and rapid intervention are crucial for improved graft survival and reducing morbidity and mortality rates [1, 2].

Biliary strictures following liver transplantation can be seen as anastomotic or non-anastomotic in nature [1, 2]. Anastomotic strictures are more common and more manageable with percutaneous and endoscopic procedures. Non-anastomotic strictures may occur following various etiologies and have poorer prognosis. Most biliary strictures are managed with endoscopic procedures. Percutaneous interventions are reserved for failed endoscopic retrograde cholangiopancreatography (ERCP) cases and for patients with altered anatomy (e.g., Roux-en-Y hepaticojejunostomy).

The success rate of endoscopic techniques in the treatment of post LDLT biliary strictures has been reported to be as 60–75% [1, 3]. Percutaneous interventions can be used alone or in combination with ERCP, with a reported clinical success rate of 50–75% [1, 3]. The overall success rate has been reported to be as high as 90% when combined ERCP and percutaneous biliary interventions are utilized [1, 3–5]. Impassable biliary obstructions are unfortunate complications and not uncommon. Magnetic compression anastomosis (MCA) is an alternative minimally invasive method of treatment for complete biliary occlusions in selected cases [6–10]. The method involves three main steps as follows: (i) insertion of magnets at cranial and caudal parts of the occlusion, (ii) waiting for complete magnetic adherence, and (iii) achieving a wire access beyond the established anastomosis (MCA) [8–12].

In this study, we aimed to investigate long-term outcomes of combined endoscopic-percutaneous methods in endoscopically unmanageable anastomotic biliary strictures encountered in LDLT recipients.

## Materials and methods

This retrospective study was approved by our institutional review board (No: SBA 23/302). The study included patients who were referred to our hospital between November 2017 and May 2023, due to biliary stricture following LDLT. Informed consent for each procedure was obtained prior to procedure for all patients. This investigation adhered to the Strengthening the Reporting of Observational Studies in Epidemiology (STROBE) guidelines [13].

### Inclusion and exclusion criteria

The main inclusion criterion was the development of biliary stricture following LDLT. The exclusion criteria were as follow: (a) age < 18 years, (b) presence of non-anastomotic biliary stricture, (c) presence of bile leakage, (d) patients’ refusal for the procedure, (e) absence of duct to duct anastomosis to common bile duct (presence of bilioenteric or cystic duct anastomoses), and (f) successful ERCP.

### Definitions, data, and procedure details

The etiology of liver disease, clinical symptoms, imaging findings and laboratory values were retrospectively obtained from the patients’ medical records. A recently published classification system was used for the discrimination of types of biliary anastomosis [14]. Complications were classified according to society of interventional radiology classification system [15].

**Fig. 1:**
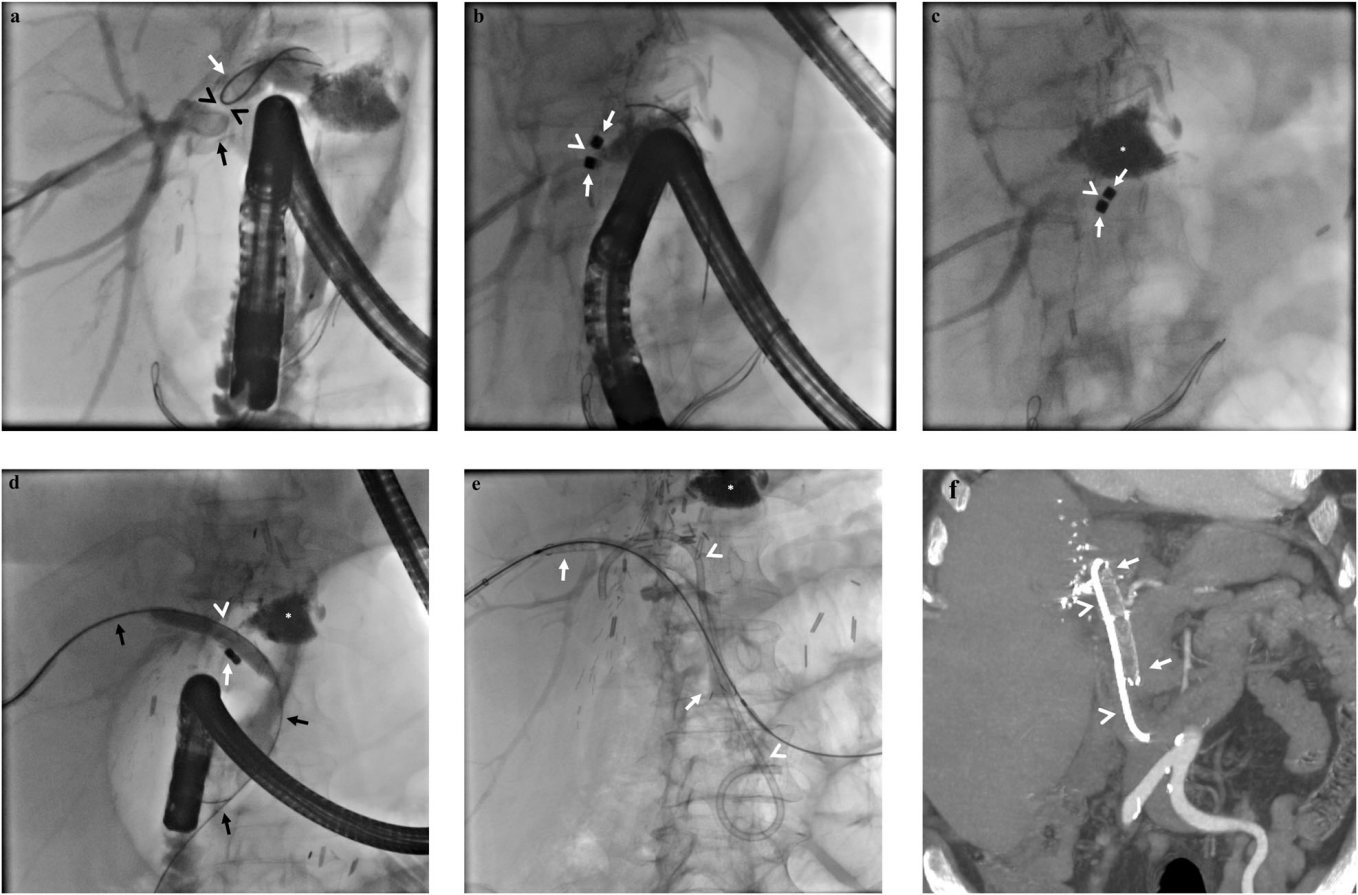
A 67-year-old woman developed biliary stricture 8 months after receiving living donor liver transplantation due to hepatitis B cirrhosis. **a** Cholangiography demonstrates complete biliary obstruction at duct-to-duct anastomosis site. Note intervening soft tissue thickness (arrowheads, a) between two wires. Both cranial (percutaneous approach, black arrow, a) and caudal (ERCP, white arrow, **a**) wire accesses had failed in traversing the obstruction, therefore magnetic compression anastomosis was decided. **b**, **c** Two magnets were pushed over the wires simultaneously via a transhepatic and ERCP approach (arrows, **b**, **c**). Immediate approach of the magnets was noted. The gap between the magnets (arrowheads, **b**, **c**) was reduced due to magnetic adherence. **d** Following complete apposition of the magnets, wire access beyond the obstruction was able to be established on the 4th day following magnet placement (black arrows, **d**). Magnets (white arrow, **d**) were pushed down to the duodenum with the support of a balloon catheter (arrowhead, **d**). **e** Finally, a fully covered metallic stent (arrows, **e**) along with a PS (arrowheads, **e**) was placed, and the percutaneous biliary drain was removed. Note metallic stent is not yet fully expanded due to stricture. **f** Coronal contrast-enhanced CT scan obtained 5 months later due to epigastric pain, revealed a fully expanded metallic stent (arrows, **f**) and PS (arrowheads, **f**). No sign of biliary dilatation is also noted. An asterisk indicates changes related to the previous vertebroplasty procedure

Percutaneous biliary drainage was performed in all patients within 24 h following a failed ERCP attempt. A 10 F internal-external biliary drainage catheter was placed in the patients for whom successful internalization (traversing the stenosis) was achieved percutaneously. Patients with severe intrahepatic biliary stones (involving at least 3 biliary ducts) were discharged with percutaneous drains, and further intervention was considered on follow-up. Patients without hepatolithiasis underwent combined endoscopic-percutaneous intervention for placement of fully-covered self-expandable metallic stent (FCSEMS) and/or plastic catheter stents (PS) prior to discharge. Following stent placement, indwelling percutaneous drains were removed in the same session. The decision for covered metallic stent (10mm × 8cm, GATE, Mediwood) placement was made depending on both percutaneous and endoscopic cholangiography findings. Covered metallic stenting was avoided in cases of more than two intrahepatic biliary ducts, given the risk for occlusion due to the meshes of the covered stent. FCSEMS was placed with a PS (7 or 10 F, double pigtail side holes placed at pigtail) to achieve additional support (FCSEMS&PS) (Fig. 1). In patients who underwent plastic biliary stenting without FCSEMS, 3 PS (one 10 F, two 7 F) were placed.

Patients for whom percutaneous internalization was unsuccessful underwent MCA. Ni-coated cylindrical neodymium iron-boron, rare-earth magnets (OBF Limited) were used in this study (3 × 4 mm, introduced via 10 F vascular sheath). All magnets had a central hole, which enabled a 0.035-inch guidewire insertion. The decision for MCA was made on cholangiography findings which was obtained during both cranial (percutaneous approach) and caudal (ERCP) wire accesses to occlusion were established (Fig. 1). MCA was avoided in case of; (i) length of stenosis exceeding 1 cm, (ii) presence of extravasation, or (iii) severe hepatolithiasis. In patients who are eligible for MCA, magnet placement was performed a few days after successful biliary drainage. Magnets were pushed over a stiff wire (Amplatz-straight tip Boston Scientific for percutaneous access, Zebra guidewire, Invamed for endoscopic access) as much as possible, as described before [7]. A guiding 5 F catheter (Imager II, Bern; Boston Scientific) and a 7-F pusher were used to push the magnet over the wire percutaneously and through the endoscope lumen, respectively. Once the magnets were placed in the desired position, a biliary drainage catheter was left (first step), and endoscopic access was removed. Then, patients were followed up with daily radiographs to observe the complete magnetic approach, which was evaluated with diminished intervening soft tissue between magnets (second step). Following complete magnetic adherence (adherence of both magnets without a residual gap), as revealed on plain radiograph, attempted wire access beyond the obstruction was performed (third step, Fig. 1). A hydrophilic guide wire (Straight tip, Terumo) was advanced percutaneously with the support of a guiding 5 F catheter to traverse the magnets. Magnets were traversed percutaneously in all patients. When the wire access beyond magnets to the duodenum was achieved, contrast injection was performed to reveal the appropriate location within the main bile duct and the duodenum. Then, a 10 F vascular sheath was introduced percutaneously through the biliary system, and both antegrade and retrograde cholangiography images were obtained. Then, the decision for covered metallic stent and/or plastic catheter stent placement was made based on cholangiography findings. Following FCSEMS and/or PS placement, patients left the operating suit without a biliary drainage catheter.

Following successful internalization with or without MCA, further step ((FCSEMS) and/or PS placement) was performed with combined endoscopic-percutaneous intervention due to several reasons as follows; (i) to be able to place multiple PS into adjacent bile ducts (no need for percutaneous access to other bile ducts), (ii) to achieve endoscopic image for final position of the stents, (iii) to be able to push a migrated PS endoscopically, and (iv) to prevent FCSEMS migration (grabbing the caudal part of the stent endoscopically by forceps) during PS insertion.

Balloon dilatation was performed in all patients prior to biliary stenting (Mustang balloon dilatation catheter 8 mm × 4 cm, Boston Scientific). The balloon was inflated to 20 atm and held in position for 1 min. Following successful MCA procedure, magnets were also pushed down to the duodenum with the same balloon catheter (Fig. 1). In case of failure (magnets are found separated from each other on daily obtained radiographs), a biopsy forceps (Olympus) was advanced percutaneously through a 10 F vascular sheath to remove dislocated magnets. All procedures were performed with a team including interventional radiologists and gastroenterologists who have at least 10 years of experience in performing percutaneous biliary intervention and ERCP, respectively.

Patients were followed up at 3-month intervals after discharge for biliary dilatation, cholangitis, and altered liver tests. All patients were evaluated with ERCP at 6 months following percutaneous biliary drain removal. Plastic and/or metallic stents were removed endoscopically, prior to retrograde cholangiography. Patients who were found to have stenosis at ERCP, were followed with stent exchange procedures and accepted as recurrence. However patients with lack of biliary obstruction findings at ERCP, were considered as success and no stent was placed.

### Statistical analyses

Categorical data were presented as numbers (percentages), and continuous variables are expressed as mean ± standard deviation (SD) or median and interquartile range (IQR) according to the distribution of the data. The 95% confidence intervals (CIs) were provided to support the follow-up data for durations that demonstrated normal distribution and were presented as mean ± SD. The normality of continuous variables was assessed using Shapiro–Wilk test. A Chi-square test was used to compare stenosis rates between MCA and non-MCA groups. A Mann–Whitney *U*-test was used to compare the stricture-free follow-up duration between the MCA and non-MCA groups. The effect size for the difference in follow-up duration between the two groups was calculated using the *r*-value, with thresholds of 0.1, 0.3, and 0.5 indicating small, medium, and large effects, respectively.

In this study, the Chi-square test was used to compare stricture rates between patients who were followed with PS only and those who were followed with FCSEMS&PS. The association between the type of stent used (PS alone or FCSEMS&PS) and restenosis was evaluated using Fisher’s exact test. This analysis was performed separately for both MCA and non-MCA groups to determine if there were significant differences in restenosis rates based on stent type. The comparison of stricture-free follow-up durations among subgroups, including percutaneously successful patients with PS, percutaneously successful patients with FCSEMS&PS, MCA successful patients with PS, and MCA successful patients with FCSEMS&PS, was performed using the Kruskal–Wallis test. Additionally, pre-procedural and post-procedural laboratory tests were compared across these subgroups using the Kruskal–Wallis test.

Two-tailed *p*-value of < 0.05 was considered statistically significant. Statistical analyses were performed using IBM SPSS Statistics (version 23, IBM SPSS).

## Results

**Fig. 2:**
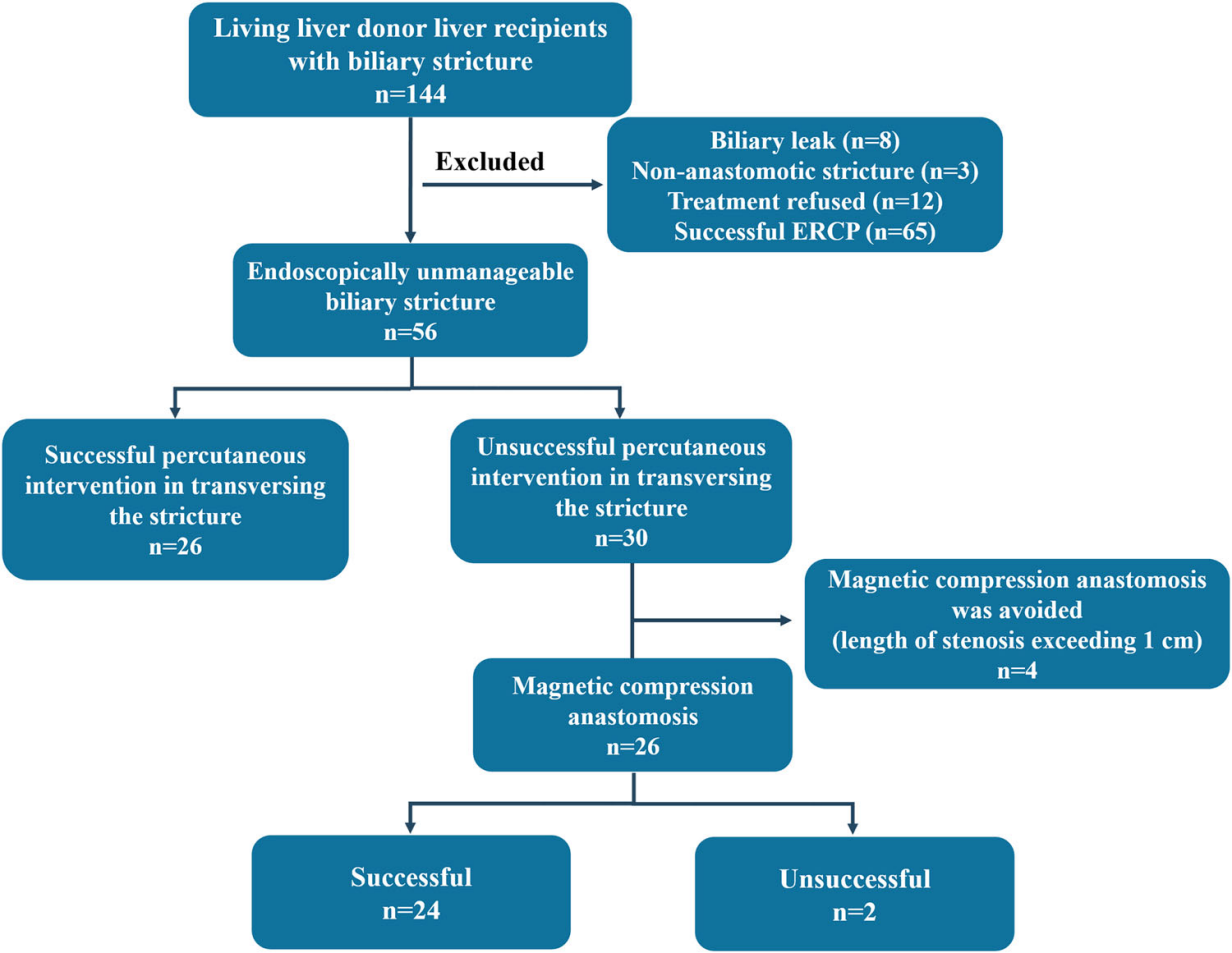
Flow diagram of the study

A total of 144 patients were referred to our hospital for biliary strictures following LDLT between November 2017 and May 2023. Eighty-eight patients (biliary leak = 8, non-anastomotic stricture = 3, treatment refused = 12, successful ERCP = 65) were excluded (Fig. 2). The final study group consisted of 56 liver transplant recipients (mean age, 59 years ± 11 (SD); *n* = 35 men) with anastomotic biliary stricture that wire access beyond the stricture could not be established endoscopically. Of the 56 patients in our study group, 35 were male (62.5%). Clinical and demographic data are presented in Table 1.

**Table 1:** Demographic and clinical characteristics in 56 patients

Parameters	Value
Sex	
Female	21 (37.5%)
Male	35 (62.5%)
Age (years)	
Female	58 ± 11
Male	59 ± 11
Overall	59 ± 11
Indications for transplantation	
Viral	20 (35.7%)
Cryptogenic	12 (21.4%)
Alcohol	11 (19.6%)
Primary biliary cirrhosis	3 (5.3%)
Non-alcoholic steatohepatitis	2 (3.6%)
Hemangioendothelioma	2 (3.6%)
Acute liver failure (drug induced)	1 (1.8%)
Caroli Disease	1 (1.8%)
Hepatocellular carcinoma	1 (1.8%)
Biliary hamartomatosis	1 (1.8%)
Autoimmune hepatitis	1 (1.8%)
Wilson disease	1 (1.8%)
Types of biliary anastomosis^a^	
Type 1	12 (21.4%)
Type 2	17 (30.4%)
Type 3	8 (14.3%)
Type 4	8 (14.3%)
Type 5	2 (3.5%)
Type 6	3 (5.3%)
Type 7b	1 (1.8%)
Type 9b	1 (1.8%)
Type 10	3 (5.3%)
Left anastomosis	1 (1.8%)
Pre-procedural liver test results	
Total bilirubin (mg/dL)	1.23 (0.79–1.89)
Direct bilirubin (mg/dL)	0.46 (0.25–0.87)
Indirect bilirubin (mg/dL)	0.72 (0.53–1.14)
Alkaline phosphatase (U/L)	343 (202–542)
Gamma-glutamyl transferase (U/L)	209 (110–329)
Post-procedural liver test results	
Total bilirubin (mg/dL)	0.84 (0.58–1.21)
Direct bilirubin (mg/dL)	0.24 (0.18–0.42)
Indirect bilirubin (mg/dL)	0.57 (0.40–0.73)
Alkaline phosphatase (U/L)	146 (118–305)
Gamma-glutamyl transferase (U/L)	76 (38–137)

^a^ Reference [14]

Percutaneous intervention was successful in traversing the biliary stricture in 26 patients (*n* = 26/56, 46%). Among these 26 patients, two patients were discharged with percutaneous biliary drainage catheters due to severe hepatolithiasis. One patient who had a previous history of recurrent cholangitis episodes was able to be discharged with a biliary drainage catheter. However he died 4 months later due to another episode of cholangitis.

There were no major adverse events related to procedures. In 23 patients, combined endoscopic-percutaneous biliary stenting was able to be performed without need for MCA. Eleven (*n* = 11/23, 47.8%) and twelve patients (*n* = 12/23, 52.2%) were discharged with multiple PS and combination of FCSEMS&PS, respectively. In all of these 23 patients, percutaneous drains were able to be removed during stent placement.

There were 30 patients (54%) in whom wire access beyond the stricture could not be achieved percutaneously. In 4 of these 30 patients, MCA was avoided due to length of stenosis (stenosis exceeding 1 cm). MCA was attempted in the remaining 26 patients (Table 2) and was successful in 24. In 2 patients, magnets failed to create anastomoses (found separated on plain radiograph) and therefore were removed. The success rate of percutaneous drain removal following MCA was 92.3% (*n* = 24/26). Time interval from initial percutaneous intervention to magnet placement and from magnet placement to internalization was 6.15 ± 2.36 days (95% CI: 5.20–7.10) and 7.71 ± 2.77 days (95% CI: 6.54–8.88), respectively.

**Table 2:** Demographic, clinical, and procedural characteristics of 26 patients in the MCA group

Parameters	Value
Sex	
Female	16 (61.5%)
Male	10 (38.5%)
Age (years)	
Female	57 ± 9
Male	62 ± 12
Overall	60 ± 11
Indication for transplantation	
Viral	10 (38.5%)
Alcohol	4 (15.4%)
Cryptogenic	3 (11.6%)
Primary biliary cirrhosis	2 (7.7%)
Non-alcoholic steatohepatitis	2 (7.7%)
Hemangioendothelioma	2 (7.7%)
Acute liver failure (drug induced)	1 (3.8%)
Hepatocellular carcinoma	1 (3.8%)
Autoimmune hepatitis	1 (3.8%)
Types of biliary anastomosis^a^	
Type 1	5 (19.2%)
Type 2	12 (46.2%)
Type 3	3 (11.6%)
Type 4	2 (7.7%)
Type 5	1 (3.8%)
Type 6	1 (3.8%)
Type 7b	1 (3.8%)
Type 10	1 (3.8%)
Pre-procedural liver test results	
Total bilirubin (mg/dL)	1.34 (0.85–1.88)
Direct bilirubin (mg/dL)	0.42 (0.27–0.87)
Indirect bilirubin (mg/dL)	0.73 (0.59–1.12)
Alkaline phosphatase (U/L)	244 (177–514.5)
Gamma-glutamyl transferase (U/L)	194 (116–236)
Post-procedural liver test results	
Total bilirubin (mg/dL)	0.86 (0.59–1.21)
Direct bilirubin (mg/dL)	0.26 (0.22–0.45)
Indirect bilirubin (mg/dL)	0.56 (0.41–0.75)
Alkaline phosphatase (U/L)	132 (107–318)
Gamma glutamyl transferase (U/L)	70 (42.5–120)
Duration from initial percutaneous intervention to magnet placement (days)	6.15 ± 2.36^b^
Duration from magnet placement to internalization (days)	7.71 ± 2.77^b^

^a^ Reference [14]

^b^ 24 patients

In 47 (*n* = 24; MCA) out of 56 patients (84%), percutaneous drains were able to be removed following successful biliary stenting. Overall follow-up was 1082.5 ± 668.2 days (95% CI: 907.49–1257.51). The median stricture-free follow-up for 47 patients was 263 (IQR: 90–720) days.

In 19 of the patients, recurrent stricture was evident at ERCP during a median follow-up of 90 (IQR: 60–210) days following stent removal. Six of these 19 patients had previously undergone MCA.

The stricture rate following MCA (*n* = 6/24, 25%) was significantly lower compared to patients in whom MCA was not required (*n* = 13/23, 56%) (*p* = 0.026). The mean stricture-free follow-up in the MCA group was 749.5 ± 670.49 days (95% CI: 466.38–1032.62). In contrast, the median stricture-free follow-up in the non-MCA group was 180 (IQR: 29–305) days. The stricture-free follow-up was statistically significantly longer in the MCA group compared to the non-MCA group (*p* = 0.006, *Z* = −2.774). A medium-sized difference (*r* = 0.40) further supports the clinical relevance of the longer follow-up without stricture in the MCA group compared to the non-MCA group.

Overall, no significant effect of FCSEMS&PS (stricture rate; *n* = 10/30, 33%) placement was found on biliary patency rate compared to PS placement alone (stricture rate; *n* = 9/17, 30%) (*p* = 0.189). Although patency rate following FCSEMS&PS placement was significantly higher (*n* = 7/12, 58%) compared to PS placement (*n* = 3/11, 27%) in the non-MCA group, it did not reach statistical significance (*p* = 0.214). In the MCA group, differences were also not found regarding the patency rate between FCSEMS&PS (patency; *n* = 13/18, 72%) and PS (patency; *n* = 5/6, 83%) groups (*p* = 1).

The stricture-free follow-up was further compared among subgroups stratified by stent types and intervention success. In the non-MCA group, the median stricture-free follow-up duration was 180 (IQR: 30–360) days for PS placement and 180 (IQR: 44.25–273.75) days for FCSEMS&PS placement. In the MCA group, the mean stricture-free follow-up duration was 1046.67 ± 910.07 days (95% CI: 91.61–2001.73) for PS placement and 650.44 ± 568.39 days (95% CI: 367.79–933.09) for FCSEMS&PS placement. A statistically significant difference in stricture-free follow-up was found among these subgroups (*p* = 0.046, *H* = 7.989). However, no significant differences were observed for pre- and post-procedure laboratory test results between these subgroups (*p* > 0.05).

Among 19 patients with recurrent stricture, ERCP was successful in traversing the stenosis in 9, however in remaining 10 patients re-percutaneous biliary access was required. Among these 10 patients, re-MCA was required in 1.

## Discussion

In this study, percutaneous biliary intervention with conventional methods yielded a success rate of 46% in traversing endoscopically unmanageable post-LDLT biliary complex stricture. In 30 patients with complete biliary occlusion, MCA was able to be performed in 26 and achieved a success rate of 92% in traversing the occluded biliary anastomosis. Overall, percutaneous access, including MCA, enabled a success rate of 84% in traversing the endoscopically unmanageable post-LDLT-biliary stricture. Percutaneous drains were removed during combined endoscopic-percutaneous biliary stenting. Follow-up ERCP findings indicated a better patency rate following MCA.

In the literature, several studies investigated the efficacy of percutaneous intervention in endoscopically unmanageable biliary strictures following LDLT and found a success rate of 85–87% in traversing the biliary stricture [3–5]. In this study, percutaneous access was able to enable wire access beyond the stricture in only 46% of the cases. In the remaining 54% of the cases, complete occlusion was found and confirmed during combined endoscopic and percutaneous cholangiography with a gap between the cranial and caudal parts of the obstruction. These findings may point out the complexity of our cases, which is also related to our low success rate. Lee et al [16] reported a success rate of 80% in percutaneously managed anastomotic biliary stricture that occurred following LDLT. They performed endoscopic-percutaneous rendezvous technique in 22 of the patients, however, there was no case of MCA. They achieved a catheter-free rate of 95.5%, however, the study group consisted of failed ERCP cases and cases in which ERCP was not attempted [16].

Our study had no appropriate cases for the rendezvous technique. Nevertheless, we argue that MCA may result in better long-term results in preserving patency compared to the rendezvous technique, in which both cranially and caudally placed wires should be extraluminal, and consequently, biliary continuity has to be disrupted. Khalaf et al [17] reported a success rate of 61% for combined endoscopic and percutaneous approach in traversing post-LDLT biliary strictures. They also stated that revision surgery was performed in failed cases (*n* = 10 patients). Our percutaneous approach failed in traversing the biliary stricture in 30 of the patients, but MCA yielded a success rate of 92% in these patients without the need for surgery, which is challenging in this patient population. Our results showed that there was no significant difference regarding patency rates between FCSEMS&PS and PS, which was in line with published literature [18, 19].

Jang et al [6] performed MCA in 12 patients with post-LDLT anastomotic biliary stenosis, in whom ERCP and percutaneous procedures had failed. They achieved successful internalization (traversing the stenosis) in 10 patients (83.3%) and found no stenosis during an average follow-up of 331 days following MCA. Li et al [20] also reported no stenosis recurrence during 2–66 months of stent-free follow-up in 9 patients who underwent MCA due to benign biliary stricture. In a different study involving 39 patients with completely obstructed benign biliary strictures, the authors reported recanalization was able to be achieved in 35 patients following MCA [21]. They also concluded that recurrence was only encountered in two patients during a mean follow-up of 41.9 months [21]. In the current study, a higher patency rate following MCA was found compared to conventional methods. We also noted that stent type had no significant effect on biliary patency between MCA and conventional methods. Jang et al [21] concluded that higher patency following MCA is due to new fistula formation instead of dilation of the previous stricture. We cannot make such a conclusion because it is beyond the scope of this study. Nevertheless, in animal-based studies regarding MCA, authors reported that full-thickness anastomosis with serosal apposition can be achieved with MCA [22, 23]. Additionally, histopathological examinations demonstrated rapid epithelization, mucosal continuity, and decreased levels of inflammation/fibrosis at the MCA site [24–26]. We consider that the presumed lack of inflammation/fibrosis and mucosal epithelial continuity through the compression anastomosis site may be the essential reasons for the expected superior patency of MCA compared to conventional methods.

There is no consensus regarding an established technique for MCA. Substantial parts of the published literature are case reports. In these reports, authors percutaneously inserted a 14–18 F sheath into biliary systems to be able to place magnets that cannot be pushed over a wire. They also waited for spontaneous anastomosis development, which was evaluated with spontaneous magnetic fall or contrast flow through the magnets on cholangiography [8–11]. In the current study, magnets were pushed over a stiff wire by the support of a guiding 5 F catheter through the lumen of a percutaneously placed a 10 F vascular sheath. Percutaneous insertion of a smaller size sheath may reduce the risk of hemorrhage and is more favorable for both the patient and operator. Another issue is the optimal timing prior to traversing the magnets. Expecting free contrast flow through magnets on cholangiography is time-consuming. Therefore, we recommend scheduling the next step, involving attempting wire access beyond the MCA, as soon as complete magnetic adherence (adherence of both magnets without a residual gap) is revealed on radiograph. Reduced time intervals between magnet placement to traversing the stenosis and utilization of small-caliber tubes may increase patient compliance with the procedure. Although magnets demonstrate a significant attractive force to each other, we observed failure of magnetic adherence in two cases, even though stricture length was < 10 mm. Such incidents have also been previously reported [20, 21]. Although the stricture length is associated with failed MCA cases, adequate magnetic power to ensure successful compression anastomosis remains unknown [20]. We postulate that patients may be advised to reduce physical activities, particularly for the 24 h following magnet placement.

Our study had several limitations. First, it was a retrospective study. Second, the number of patients was low in subgroups; therefore, strong statistical evidence could not be obtained. Third, there was a variety among the transplantation etiologies. Fourth, we could not acquire any data on radiation exposure during the procedure due to the retrospective nature of our study. A retrospective study design with a low patient population in subgroups may have been affected by patient selection bias. However, it should be noted that MCA was performed for the patients in whom other attempts had failed in traversing the biliary obstruction. Moreover, patients who underwent MCA had also previously failed attempts at ERCP and had complex occlusions. This study was a report of a tertiary referring care center. Patients were referred from different hospitals due to the failure of wire access beyond the duct-to-duct biliary anastomoses. Our unexpectedly low success rate in traversing a post-transplant biliary obstruction (almost 50%) compared to published literature is primarily related to the above-mentioned challenges. Previous attempts, which may also be complicated with extravasation, hemorrhage, and/or edema, can also be held responsible for the low success rate of our percutaneous attempts. Therefore, one may argue about patient selection bias between subgroups. Nevertheless, this study was conducted to investigate long-term outcomes of the combined endoscopic-percutaneous approach in these complex biliary obstructions encountered in LDLT recipients. Despite complex biliary obstructions in the MCA group, magnetic compression anastomosis yielded superior recurrence-free follow-up.

In conclusion, percutaneous biliary procedures are essential for endoscopically unmanageable anastomotic biliary strictures in living donor liver recipients. Combined endoscopic and percutaneous interventions enable MCA procedure which may obviate the need for revision surgery. MCA seems to provide higher patency rates even in patients with total biliary occlusion. Data reported in this study revealed that stent type had not effect on patency rate.

## Abbreviations

ERCP: Endoscopic retrograde cholangiopancreatography
FCSEMS: Fully covered self-expandable metallic stent
LDLT: Living donor liver transplantation
MCA: Magnetic compression anastomosis
PS: Plastic catheter stents

## References

[1] Magro B, Tacelli M, Mazzola A, Conti F, Celsa C (2021) Biliary complications after liver transplantation: current perspectives and future strategies. Hepatobiliary Surg Nutr 10:76–92 33575291 10.21037/hbsn.2019.09.01 PMC7867735

[2] Boeva I, Karagyozov PI, Tishkov I (2021) Post-liver transplant biliary complications: Current knowledge and therapeutic advances. World J Hepatol 13:66–79 33584987 10.4254/wjh.v13.i1.66 PMC7856868

[3] Karatoprak S, Kutlu R, Karatoprak NB, Dag N, Yilmaz S (2021) Percutaneous radiological biliary interventions after failed endoscopic treatment in living liver donors: experience of a high-volume transplantation center. Transpl Int 34:2846–2855 34559926 10.1111/tri.14118

[4] Kim ES, Lee BJ, Won JY, Choi JY, Lee DK (2009) Percutaneous transhepatic biliary drainage may serve as a successful rescue procedure in failed cases of endoscopic therapy for a post-living donor liver transplantation biliary stricture. Gastrointest Endosc 69:38–46 18635177 10.1016/j.gie.2008.03.1113

[5] Jegadeesan M, Goyal N, Rastogi H, Gupta S (2019) Percutaneous transhepatic biliary drainage for biliary stricture after endotherapy failure in living donor liver transplantation: a single-centre experience from India. J Clin Exp Hepatol 9:684–689 31889748 10.1016/j.jceh.2019.03.004 PMC6926189

[6] Jang SI, Cho JH, Lee DK (2020) Magnetic compression anastomosis for the treatment of post-transplant biliary stricture. Clin Endosc 53:266–275 32506893 10.5946/ce.2020.095 PMC7280848

[7] Ünal E, Çiftçi TT, Akinci D et al (2024) Magnets in action: role of interventional radiologists in magnetic compression anastomosis procedures. Insights Imaging 15:128. 10.1186/s13244-024-01705-9 38816640 10.1186/s13244-024-01705-9 PMC11139847

[8] Takao S, Matsuo Y, Shinchi H et al (2001) Magnetic compression anastomosis for benign obstruction of the common bile duct. Endoscopy 33:988–990 11668410 10.1055/s-2001-17923

[9] Akita H, Hikita H, Yamanouchi E et al (2008) Use of a metallic-wall stent in the magnet compression anastomosis technique for bile duct obstruction after liver transplantation. Liver Transpl 14:118–120 18161766 10.1002/lt.21273

[10] Matsuno N, Uchiyama M, Nakamura Y et al (2009) A nonsuture anastomosis using magnetic compression for biliary stricture after living donor liver transplantation. Hepatogastroenterology 56:47–49 19453026

[11] Okajima H, Kotera A, Takeichi T et al (2005) Magnet compression anastomosis for bile duct stenosis after duct-to-duct biliary reconstruction in living donor liver transplantation. Liver Transpl 11:473–475 15776404 10.1002/lt.20364

[12] Suyama K, Takamori H, Yamanouchi E et al (2010) Recanalization of obstructed choledochojejunostomy using the magnet compression anastomosis technique. Am J Gastroenterol 105:230–231 20054323 10.1038/ajg.2009.473

[13] von Elm E, Altman DG, Egger M et al (2007) The Strengthening the Reporting of Observational Studies in Epidemiology (STROBE) statement: guidelines for reporting observational studies. Lancet 370:1453–1457 18064739 10.1016/S0140-6736(07)61602-X

[14] Parlak E, Simsek C, Koksal AS et al (2022) The classification of biliary strictures in patients with right-lobe liver transplant recipients and its relation to traversing the stricture with a guidewire. Transplantation 106:328–336 33724243 10.1097/TP.0000000000003738

[15] Baerlocher MO, Nikolic B, Sze DY (2023) Adverse event classification: clarification and validation of the society of interventional radiology specialty-specific system. J Vasc Interv Radiol 34:1–3 36244632 10.1016/j.jvir.2022.10.011

[16] Lee IJ, Lee JH, Kim SH et al (2022) Percutaneous transhepatic treatment for biliary stricture after duct-to-duct biliary anastomosis in living donor liver transplantation: a 9-year single-center experience. Eur Radiol 32:2414–2425 35064314 10.1007/s00330-021-08373-z

[17] Khalaf H, Alawi K, Alsuhaibani H et al (2011) Surgical management of biliary complications following living donor liver transplantation. Clin Transplant 25:504–510 21070364 10.1111/j.1399-0012.2010.01338.x

[18] Martins FP, De Paulo GA, Contini MLC, Ferrari AP (2018) Metal versus plastic stents for anastomotic biliary strictures after liver transplantation: a randomized controlled trial. Gastrointest Endosc 87:131.e131–131.e113 10.1016/j.gie.2017.04.013 28455159

[19] Visconti TAC, Bernardo WM, Moura DTH et al (2018) Metallic vs plastic stents to treat biliary stricture after liver transplantation: a systematic review and meta-analysis based on randomized trials. Endosc Int Open 6:E914–E923 30258982 10.1055/a-0626-7048 PMC6156748

[20] Li Y, Sun H, Yan X et al (2020) Magnetic compression anastomosis for the treatment of benign biliary strictures: a clinical study from China. Surg Endosc 34:2541–2550 31399950 10.1007/s00464-019-07063-8

[21] Jang SI, Lee KH, Yoon HJ, Lee DK (2017) Treatment of completely obstructed benign biliary strictures with magnetic compression anastomosis: follow-up results after recanalization. Gastrointest Endosc 85:1057–1066 27619787 10.1016/j.gie.2016.08.047

[22] Gonzales KD, Douglas G, Pichakron KO et al (2012) Magnamosis III: delivery of a magnetic compression anastomosis device using minimally invasive endoscopic techniques. J Pediatr Surg 47:1291–1295 22703808 10.1016/j.jpedsurg.2012.03.042

[23] Jamshidi R, Stephenson JT, Clay JG, Pichakron KO, Harrison MR (2009) Magnamosis: magnetic compression anastomosis with comparison to suture and staple techniques. J Pediatr Surg 44:222–228 19159747 10.1016/j.jpedsurg.2008.10.044

[24] Parlak E, Eminler AT, Koksal AS et al (2019) A new method for lumen restoration in a patient with aphagia: Oro-oesophageal through-the-scope magnetic compression anastomosis. Clin Otolaryngol 44:1214–1217 30968566 10.1111/coa.13337

[25] Ore AS, Althoff A, Kull DR, Baldwin TJ, Van Eps JL, Messaris E (2023) Comparative early histologic healing quality of magnetic versus stapled small bowel anastomosis. Surgery 173:1060–1065 36566103 10.1016/j.surg.2022.11.030

[26] Ryou M, Agoston AT, Thompson CC (2016) Endoscopic intestinal bypass creation by using self-assembling magnets in a porcine model. Gastrointest Endosc 83:821–825 26522371 10.1016/j.gie.2015.10.023

